# Integrated bioinformatics and mendelian randomization reveal a six-gene diagnostic signature and key role of CYP26B1 in sarcopenia

**DOI:** 10.3389/fmolb.2026.1760938

**Published:** 2026-02-26

**Authors:** Yaoqi Wu, Xiaoqing Cai, Shiwen Fan, Lina Zhao, Yingying Jiao, Tongkai Chen, Manting Liu, Yafang Song

**Affiliations:** 1 Science and Technology Innovation Center, Guangzhou University of Chinese Medicine, Guangzhou, Guangdong, China; 2 Department of Gastrosplenic Diseases, The First Affiliated Hospital of Guangzhou University of Chinese Medicine, Guangzhou, Guangdong, China

**Keywords:** bioinformatics, diagnostic biomarker, machine learning, mendelian randomization, sarcopenia

## Abstract

**Background:**

The pathogenesis of sarcopenia involves complex molecular mechanisms, and treatment remains challenging, with a lack of reliable diagnostic biomarkers. The objective of this study is to identify biomarkers that may be linked to sarcopenia, examine how these biomarkers correlate with immune cell infiltration, and investigate the genes that exhibit a causal relationship with sarcopenia.

**Methods:**

Four transcriptomic datasets were integrated to identify candidate biomarkers. Genes from the MEBrown module of weighted gene co-expression network analysis (WGCNA) analysis were cross-referenced with differentially expressed genes (DEGs). A diagnostic model was built using 113 machine learning algorithms, followed by protein-protein interaction (PPI) network analysis and SHapley Additive exPlanations (SHAP) evaluation. Immune cell quantification and correlation with sarcopenia-related genes were performed using CIBERSORT, while gene expression data was integrated with genome-wide association statistics (GWAS) and gene expression quantitative trait loci (eQTL) data. *In vitro* validation was carried out using C2C12 cells and quantitative polymerase chain reaction (qPCR) experiments.

**Results:**

We found 318 DEGs. By comparing the WGCNA gene with these DEGs, we found 109 possible biomarkers, which are related to immune regulation, muscle cytoskeleton regulation and retinol metabolism. A six-gene diagnostic signature (FOXO1, ZBTB16, HOXB2, LYVE1, MGP, and CYP26B1) was developed using machine learning and PPI network analysis, achieving high predictive accuracy (AUC >0.80), with HOXB2 identified as the top predictor via SHAP analysis. CIBERSORT analysis showed the relationship between these genes and immune cell subsets, while Mendelian randomization (MR) analysis confirmed the causal relationship between the expression of CYP26B1 gene and the risk of sarcopenia. The result of qPCR analysis is the same as the mRNA expression found in Gene Expression Omnibus (GEO) data set.

**Conclusion:**

This study identified a highly reliable six-gene diagnostic signature for sarcopenia. Mendelian randomization established CYP26B1 as the sole causal factor, linking retinoic acid metabolism to disease etiology. This dual evidence provides a robust six-gene diagnostic model and a prioritized therapeutic target, elucidating immune-metabolic mechanisms of sarcopenia. These findings offer new avenues for early diagnosis and metabolism-based precision therapy.

## 1 Introduction

Sarcopenia is an age-related systemic muscle disease characterized by pathological declines in muscle mass, muscle strength, and gait speed (Cruz-Jentoft and Sayer, 2019; Sayer et al., 2024). It is highly prevalent among older adults and significantly increases the risk of falls, disability, and mortality, thereby severely impairing quality of life (Wakabayashi and Sakuma, 2014). Epidemiological research indicates that around 10%–16% of the global elderly population is affected by sarcopenia (Yuan and Larsson, 2023; Kalra et al., 2025).

The pathogenesis of sarcopenia is complex and involves multiple molecular and cellular mechanisms, including alterations in muscle fiber composition, hormonal dysregulation, impaired satellite cell function, neuromuscular junction dysfunction, protein homeostasis imbalance, chronic inflammation, and mitochondrial dysfunction (Wiedmer et al., 2021). However, these mechanisms are still not fully understood (Cannataro et al., 2021). Pathologically, sarcopenia is characterized by a reduction in both the number and size of muscle fibers, particularly type II fibers, accompanied by fat infiltration into skeletal muscle tissue (Cruz-Jentoft and Sayer, 2019).

Currently, nutritional supplementation and resistance exercise are the main therapeutic strategies for sarcopenia, and no pharmacological treatment has yet been approved (Sakuma et al., 2023; Chung et al., 2025). Nevertheless, these methods often face issues of slow therapeutic response and poor patient compliance, highlighting the urgent need for precise new strategies targeting the pathological mechanisms (Cho et al., 2022). In clinical practice, muscle mass is commonly evaluated using bioelectrical impedance analysis (BIA) and dual-energy X-ray absorptiometry (DXA). Nevertheless, the high cost of equipment, technical requirements, and susceptibility to physiological factors such as hydration status limit their widespread application (Papadopoulou, 2020; Cho et al., 2022). Early diagnosis is therefore critical, as timely intervention may delay or prevent disease progression (Cheng J. P. et al., 2025). These limitations underscore the importance of identifying reliable molecular biomarkers for sarcopenia.

We found transcriptome data related to muscle tissue from Gene Expression Omnibus (GEO) database, which included samples provided by people diagnosed as sarcopenia and healthy people.There are four expression profiles: GSE1428, GSE8479, GSE136344 and GSE111016. We mainly analyzed GSE1428 and GSE8479, and the test data sets were GSE136344 and GSE111016. Differentially expressed genes between sarcopenia and control samples were first identified. Weighted gene co-expression network analysis (WGCNA) was then applied to construct co-expression modules and screen key functional gene clusters. Overlapping genes between the significant modules and differentially expressed genes were regarded as candidate biomarkers and further subjected to Gene Ontology (GO) and Kyoto Encyclopedia of Genes and Genomes (KEGG) enrichment analyses.To identify hub diagnostic genes, we integrated machine learning algorithms with protein–protein interaction (PPI) network analysis and constructed a robust diagnostic model, which was interpreted using SHapley Additive exPlanations (SHAP). The association between hub genes and immune cell infiltration was investigated using CIBERSORT. Furthermore, Mendelian randomization (MR) analysis based on genome-wide association studies (GWAS) and expression quantitative trait loci (eQTL) data was performed to explore potential causal relationships between key genes and sarcopenia. Finally, *in vitro* experiments were conducted to validate the bioinformatics findings.

## 2 Methods

### 2.1 Research design

**FIGURE 1:**
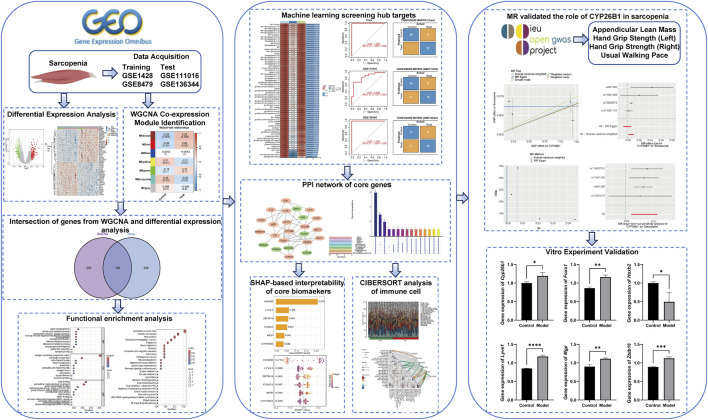
Graphical abstract illustrating the overall workflow for identifying and causally validating diagnostic biomarkers of sarcopenia based on transcriptomic integration, machine learning, and Mendelian randomization.

In this study, we used GEO database (https://www.ncbi.nlm.nih.gov/geo/) to find the genes related to sarcopenia by differentially expressed genes (DEGs) and WGCNA analysis. Then we further screened out the core genes by machine learning and PPI network analysis. SHAP algorithm helps us to explain the diagnostic model of sarcopenia. At the same time, we also combine the GWAS data about sarcopenia with the eQTL data of specific genes to find out the possible causal relationship between key genes and sarcopenia. We also established a model of sarcopenia with C2C12 cells, so that we can verify the key genes through quantitative polymerase chain reaction (qPCR) experiments *in vitro*.The overall study design and analytical workflow are summarized in Figure 1, which was constructed as a graphical abstract to visually illustrate the integrated research framework, following the principles of effective graphical abstract design (Kamble et al., 2025).

### 2.2 Data collection and analysis

We got the data set about sarcopenia from GEO database. There are four data sets that can be used publicly: GSE1428 (Giresi et al., 2005), GSE8479 (Melov et al., 2007), GSE136344 (Gueugneau et al., 2021) and GSE111016 (Migliavacca et al., 2019). In this study, we selected GSE1428 and GSE8479 as training data sets, which included 37 sarcopenia samples (12 from GSE1428 and 25 from GSE8479) and 36 control muscle samples (10 from GSE1428 and 26 from GSE8479). In order to deal with the batch effect and ensure the consistency of data, we first use the “SVA” toolkit of R language to correct the batch effect. After that, we combined the arrays of each group and used principal component analysis (PCA) to reduce the dimension and the possible batch effect. Then, we use R’s “Limma” toolkit to analyze the differential expression of the integrated data set to find those DEGs that meet the conditions of |logFC| > 0.5 and *P.adjust* <0.05. Finally, we use the “Pheatmap” toolkit to draw these DEGs in the form of heat maps. Because all the data sets we use are public, this study does not need to be approved by the ethics Committee.

### 2.3 Weighted gene Co-expression network analysis of DEGs

WGCNA constructed a scale-free network by linking the gene expression level with clinical features (Langfelder and Horvath, 2008). In this study, we used the “WGCNA” of R package to find the gene module which is particularly closely related to sarcopenia. First of all, we choose the most suitable soft threshold to build a network structure that conforms to the scale-free distribution. Then, we build a weighted adjacency matrix, and then turn it into a topological overlap matrix (TOM). After that, we calculated the difference between genes according to TOM, and grouped the genes by dynamic tree cutting algorithm, so that we could find out all kinds of co-expression modules. Finally, we chose the gene module which is most related to the phenotype of sarcopenia for further analysis.

### 2.4 GO and KEGG enrichment analysis of key genes

GO enrichment analysis is a commonly used bioinformatics method, which divides the functional description of genes into three different types: molecular function, biological process and cellular component (Aleksander et al., 2023). Similarly, KEGG pathway enrichment analysis is often used to study the biological mechanism and function of genes (Kanehisa and Goto, 2000). These descriptions cover many fields, such as human diseases, biological processes, and signal pathways, which can help us find out the possible connections between genes related to sarcopenia and let us better understand how they lead to the development of this disease. Based on this, we conducted a comprehensive GO functional analysis and KEGG pathway enrichment analysis on the common genes, using the two toolkits of “clusterProfiler” and “DOSE” in R language. We set an important standard for these analyses, that is, the p value should be less than 0.05 to be meaningful. Finally, we use the “GOplot” toolkit to draw a circular diagram to show these results.

### 2.5 Constructing the optimal model using integrated machine learning algorithms

In the R language environment, we use 12 different algorithms for variable selection and model building, including ElasticNet, Stepglm, Lasso, Generalized Linear Model Boosting (glmBoost), Ridge, Elastic Net (Enet), Support Vector Machine (SVM), Partial Least Squares Regression for Generalized Linear Models (plsRglm), Linear Discriminant Analysis (LDA), Random Forest (RF), NaiveBayes and eXtreme Gradient Boosting (XGBoost) (Liang et al., 2025). This method combines linear regression model with advanced nonlinear learning method, which can give us a lot of analytical inspiration. The whole calculation framework adopts double algorithm strategy: (1) Firstly, the prediction factors are selected by feature sorting through recursive feature elimination, and then the prediction model is established by stacking generalization technology. (2) This method runs under the framework of hierarchical 10-fold cross-validation, and 113 different configurations are generated by system optimization superparameter (Li T. et al., 2025). Then, we calculated the consistency index (C-Index) of all combinations with the external verification set, and visualized the results through the heat map for evaluation (Zhang W. et al., 2025). Based on GSE1428 and GSE8479 data sets, we use the combination of 113 algorithms mentioned above to build a diagnosis model. In addition, the model is externally verified on two different data sets, GSE136344 and GSE111016.Finally, the model achieving the highest average area under the curve (AUC) across both the training and test sets was selected as the optimal model for this study.

### 2.6 Construction of protein-protein interaction network

A PPI network was established utilizing information obtained from the STRING database to depict the interconnections among various proteins (Szklarczyk et al., 2019). This network specifically highlights the overlap between DEGs and the genes identified through the WGCNA analysis. Using the Cytoscape softwareplugin “Cytohubba” (Chin et al., 2014) the top 15 genes were selected based on their scores in various network centrality measures, including MCC, EPC, Degree, MNC, EcCentricity, DMNC, Radiality, Bottle Neck, Closeness, Betweenness, Clustering Coefficient, and Stress.The core genes of Cytohubba were then identified from these genes.

### 2.7 Diagnostic ability and expression levels of core genes

In order to assess the diagnostic potential and expression levels of critical genes associated with sarcopenia, we employed the R package “pROC” to generate receiver operating characteristic (ROC) curves. This method helps us to calculate the AUC, and we use this AUC to evaluate the classification ability of each gene in distinguishing the people with sarcopenia from the control group. In the training and verification data set, as long as the AUC value of genes exceeds 0.7, we think that they have a good diagnostic effect, indicating that they may become biomarkers of sarcopenia. In addition, the R package “PerformanceAnalytics” was applied to analyze and visualize the correlations among the key genes in the validation dataset.

### 2.8 Model interpretation

To improve the transparency and interpretability of the model, the SHAP algorithm was applied to explain the sarcopenia diagnostic model. This method will score each feature and calculate the SHAP value of each feature, so that we can understand how each feature affects the prediction results of the model (Zhang M. et al., 2025).

### 2.9 CIBERSORT analysis of immune cell subtypes related to sarcopenia

The “CIBERSORT” package was employed to evaluate the degree of immune cell infiltration within the gene expression dataset associated with sarcopenia.In order to show the number and distribution of immune cells in different samples, we draw a histogram with the tool of “ggplot2”. Then, we use the R toolkit ggpubr to compare the proportion of 22 different immune cells in sarcopenia samples and normal control samples, and use Wilcoxon test for statistical analysis. The analysis results are displayed by the stack histogram made by ggplot2. In addition, we also use the “corrplot” toolkit to draw the correlation between 22 kinds of immune cells. When the p value is less than 0.05, we think that the correlation is significant.

### 2.10 MR analysis for key gene prioritization

MR is conducted to verify whether the direction of effects observed in the differential gene expression model aligns with the results from the MR approach.The exposure data and gene eQTL data we used were all obtained from GWAS summary data. As for the results related to sarcopenia-such as lean body weight of limbs, grip strength of hands, and walking speed-they are all from UK Biobank, and only use the information of European participants (Supplementary Table S1). In MR analysis, we regard the gene expression level related to sarcopenia as the exposure factor, while the gene variation, especially single nucleotide polymorphisms (SNPs) in eQTL data set, is used as a tool variable to study their possible causal relationship with the risk of sarcopenia. In order to ensure the reliability of the tool, we only consider those genes with at least three effective SNPs. We will evaluate the strength of these tool variables by calculating the F statistics of each SNP set. If the F statistic is greater than 10, it means that it is a strong tool variable, which can help reduce the possible bias in MR analysis.

When we do MR analysis, we mainly use the method of Inverse Variance Weighted (IVW). At the same time, we also use other methods to help, such as MR-Egger, weighted median, simple mode and weighted mode. In order to ensure the reliability of the results, we will carry out pleiotropic test, heterogeneity evaluation and sensitivity analysis. The method of sensitivity analysis is to remove one data at a time and then see if the results will change. The screening criteria we set are: the P value of IVW should be less than 0.05, the odds ratios (OR) obtained by different MR methods should be consistent, and the P value of pleiotropic test should be greater than 0.05. In order to improve the accuracy and reliability of MR analysis, we will use MR-PRESSO to find out the biased SNPs and then remove them. We evaluate the heterogeneity by IVW and MR-Egger test. If the p value is greater than 0.05, it means that there is no obvious heterogeneity in the data. In addition, we use MR-Egger intercept test to check pleiotropy. If the p value is greater than 0.05, it means that there is no pleiotropic effect. For those genes that meet all these criteria, we will use various graphs for further analysis and display, such as forest plots, scatter plots, funnel plots and leave-one-out analysis, so that we can see the causal relationship more clearly.

### 2.11 Quantitative PCR verification

Mouse C2C12 myoblasts were originally obtained from the Cell Bank of Chinese Academy of Sciences (CBCC) and procured for this study from Jinyuan Biotechnology (Shanghai, China).Cells were divided into Control and Model groups. To establish an *in vitro* sarcopenia model, cells in the Model group were treated with 100 μM hydrogen peroxide (H_2_O_2_) for 24 h, while the Control group was cultured under identical conditions without H_2_O_2_ (Feng et al., 2022; Wu X. et al., 2025). After treatment, total RNA was extracted from the C2C12 cells using the standard TRIzol method. The purity and concentration of extracted RNA were measured by NanoDrop spectrophotometer. According to the method of reverse transcription kit, we reverse transcription a microgram of RNA and made a cDNA.qPCR was performed on an ABI 7500 Real-Time PCR System using SYBR Green Master Mix. The expression levels of the target genes were normalized to β-Actin as the internal reference gene. The primer sequences used in this study are listed in Table 1.

**TABLE 1:** Primer sequences used for real-time qPCR.

Gene	Species	Orientation	Primer sequences (5′–3′)	Product size (bp)
*Cyp26b1*	*Mus muslcuhs*	Forward Reverse	GGTTTCCAGATCCCCAAGGG GCCAAACGGGAGGTAATGGA	159
*Hoxb2*	*Mus muslcuhs*	Forward Reverse	CGAGGTCGGATCACCATCAG TTCTCCAGCTCCAGCAGTTG	108
*Lyve1*	*Mus muslcuhs*	Forward Reverse	GACACTCAAACACCCGCAAC TGGTGGCAGAAACAGGTGTT	91
*Foxo1*	*Mus muscuhs*	Forward Reverse	TCGCCACAATCTGTCCCTTC TTCTCCGGGGTGATTTTCCG	119
*Zbtb16*	*Mus muslcuhs*	Forward Reverse	CAGAGGGAGCTGTTCAGCAA CACTGTGCAGTTTCCTGTGC	142
*Mgp*	*Mus muslcuhs*	Forward Reverse	GAGAGTCCAGGAACGCAACA GCGTTGTAGCCGTAGACCAT	102
*β-actin*	*Mus muslcuhs*	Forward Reverse	GATGGTGGGAATGGGTCAGAAGG TTGTAGAAGGTGTGGTGCCAGATC	147

### 2.12 Statistical analysis

We use R software (version 4.5.1) and GraphPad Prism for statistics. Continuous variables are presented as mean ± standard deviation, while categorical variables are reported as frequencies and percentages. Using the method of T-test, we compared the mRNA expression levels between different groups.To evaluate the diagnostic accuracy of the candidate mRNA, a ROC curve was plotted, and the AUC was determined through the application of a logistic regression model. Additionally, sensitivity and specificity at the optimal cutoff value were determined. The ROC curve was constructed by plotting sensitivity against (100-specificity), with the results expressed as the AUC and its 95% confidence interval. All statistical evaluations were performed utilizing the R programming language.

## 3 Results

### 3.1 DEGs were identified

**FIGURE 2:**
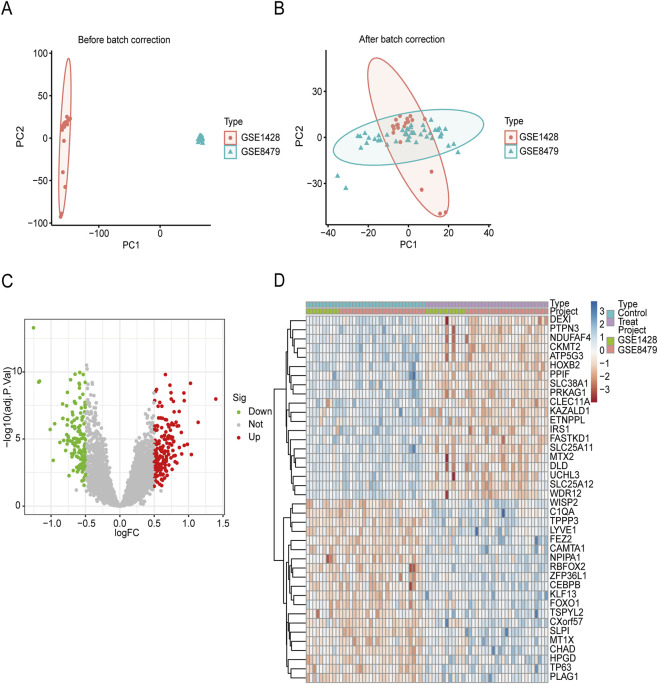
DEG between healthy controls and sarcopenia. **(A)** The application of PCA on the two datasets was performed without any prior normalization; **(B)** Subsequently, PCA was conducted on the first and second principal components (PC1 and PC2) after normalizing the data derived from the two datasets; **(C)** A volcano plot illustrating the DEGs between the control and sarcopenia groups; **(D)** A heatmap displaying the top 40 DEGs identified between the control and sarcopenia groups. DEGs, differentially expressed genes; PCA, principal component analysis; PC1, principal component 1; PC2, principal component 2.

The GSE1428 and GSE8479 microarray datasets were subjected to normalization and batch correction, and subsequently merged to form a larger cohort for both training and internal validation purposes. After the normalization and batch correction processes, PCA was performed, demonstrating successful integration of the samples from both datasets, which confirmed effective harmonization across platforms (Figures 2A,B). Using the limma package with criteria of FDR <0.05 and |Log2 FC| > 0.5, we identified a total of 318 DEGs (Supplementary Table S2) when comparing the sarcopenia patient cohort to the healthy control group (Figures 2C,D).

### 3.2 Assessment of key modules in the weighted gene co-expression network

**FIGURE 3:**
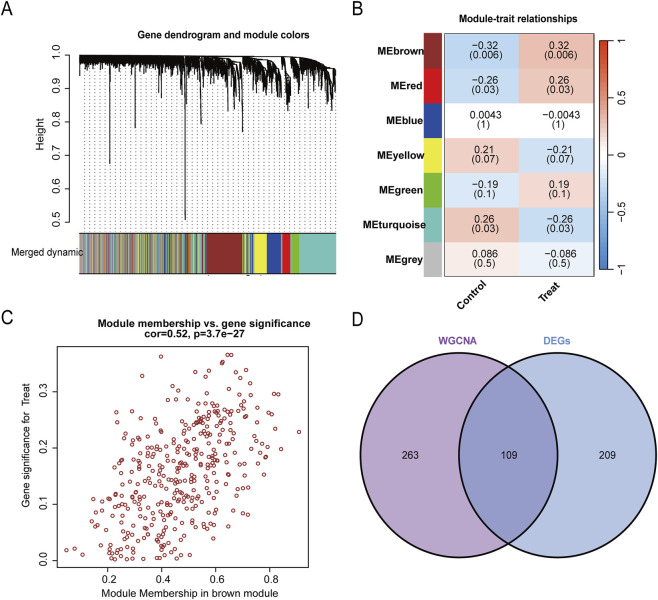
WGCNA screens immune-related genes. **(A)** Thresholds for WGCNA analysis. **(B)** Module-feature relationships. Each cell contains the corresponding correlation and p-value. **(C)** Scatter plot illustrating the correlation between module membership (MM) and gene significance (GS) within the brown module. **(D)** Venn diagram representing the overlap between DEGs and genes within the brown module.

In order to systematically identify key genes associated with the sarcopenia phenotype,we undertook an assessment of the weighted gene co-expression network. As illustrated in Figure 3, the MEbrown module exhibited the most pronounced negative correlation with sarcopenia within the studied cohort (correlation = −0.32, *P* = 0.006, refer to Figures 3A,B). Additionally, we observed a statistically significant variation in the distribution of gene significance (GS) across various modules (*P* = 0.05). Subsequently, we conducted an intra-module analysis, which uncovered a significant positive correlation between module membership (MM) and GS specifically in the MEbrown module (correlation = 0.52, *P* = 3.7e−27) (Figure 3C). Finally, we intersected the 372 genes in the MEbrown module with the previously identified DEGs, resulting in the selection of 109 candidate genes for further machine learning analysis (Figure 3D).

### 3.3 Functional enrichment analysis

**FIGURE 4:**
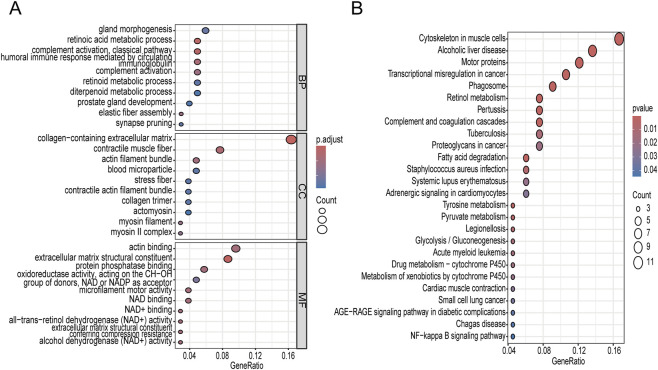
Functional enrichment analysis of intersecting genes in GO and KEGG. **(A)** Pie chart of GO enrichment functions; **(B)** Bubble chart of KEGG signaling pathways. GO, Gene Ontology; KEGG, Kyoto Encyclopedia of Genes and Genomes; DEGs, differentially expressed genes.

GO and KEGG enrichment analyses were performed on the genes identified as common across the datasets. The GO enrichment results showed that these genes were mainly involved in gland morphogenesis, retinoic acid metabolic process, complement activation, and humoral immune response in the Biological Process (BP) category, suggesting their potential roles in tissue development, immune regulation, and retinoid metabolism (Figure 4A). In the Cellular Component (CC) category, the significantly enriched terms included collagen-containing extracellular matrix, contractile muscle fiber, actin filament bundle, and myosin filament, indicating that DEGs are closely associated with cytoskeletal organization and extracellular matrix remodeling. In the Molecular Function (MF) category, extracellular matrix structural constituent, actin binding, oxidoreductase activity, and NAD/NAD^+^ binding were prominently enriched, implying the involvement of these genes in structural maintenance, cytoskeletal dynamics, and redox-related metabolic processes. Furthermore, KEGG pathway analysis demonstrated that DEGs were significantly enriched in pathways such as cytoskeleton in muscle cells, transcriptional misregulation in cancer, complement and coagulation cascades, phagosome, and retinol metabolism (Figure 4B). These pathways suggest that the dysregulated genes may participate in cytoskeletal reorganization, immune and inflammatory responses, metabolic reprogramming, collectively highlighting the potential roles of DEGs in extracellular matrix remodeling, immune modulation, and metabolic alterations underlying the studied condition.

### 3.4 Constructing a sarcopenia diagnostic model through machine learning

**FIGURE 5:**
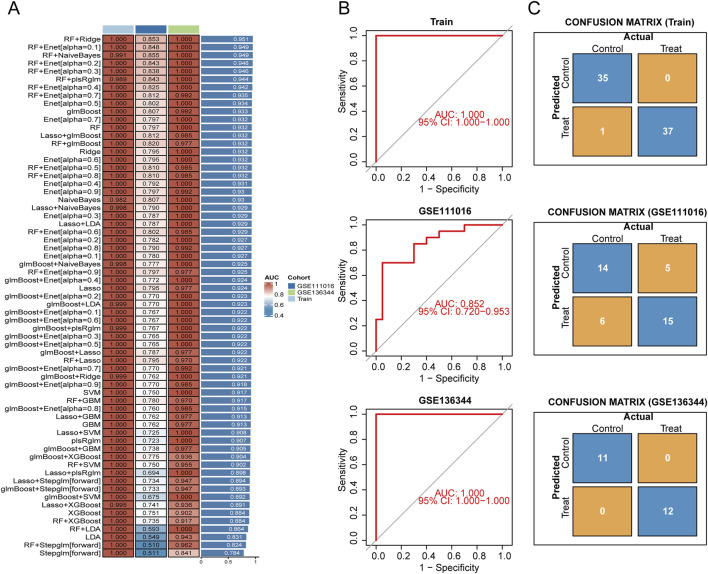
Development and assessment of the feature gene model utilizing machine learning techniques. **(A)** The heatmap illustrates the predictive performance, represented by the AUC, of 113 machine learning combinations evaluated on the training dataset (Train) as well as two validation datasets (GSE111016, GSE136344). **(B)** The ROC curves for the chosen model are depicted for both the training dataset and the validation datasets, demonstrating its ability to classify effectively. **(C)** Confusion matrix visualizations for the training and validation datasets are presented.

For the 109 potential key genes identified earlier, leading to the generation of 113 distinct combinations utilizing 12 varied machine learning algorithms aimed at determining the most dependable diagnostic model. To ensure the robustness of our findings, we extended this comprehensive analysis beyond the initial training model to include two independent external validation datasets (Figure 5A). We assessed the utility of each feature selection technique by strictly monitoring the classification accuracy of the resulting models on the validation set. For the internal training dataset, we applied a 10-fold cross-validation strategy across every algorithmic combination to compute the associated AUC values. As detailed in the rankings presented in Figure 5A, the pairing of RF with Ridge regression emerged as the clear standout. This combination demonstrated superior predictive capability across both the internal and external cohorts, achieving AUC values of 1.000 (95% CI: 1.000–1.000), 0.852 (95% CI: 0.720–0.953), and 1.000 (95% CI: 1.000–1.000), respectively (Figure 5B). The confusion matrix shown in Figure 5C further indicates that the model achieved an impressive overall accuracy of 98.6% on the training set, calculated as (35 + 37)/(35 + 0+1 + 37). The model exhibited strong classification performance across all datasets, demonstrating high predictive accuracy (Figure 5C). The RF + Ridge algorithm identified 25 hub genes (HOXB2, SLPI, TPPP3, PLAG1, COL4A5, PPIF, KLF13, LYVE1, FOXO1, ST8SIA5, TMEM158, KAZALD1, ZBTB16, FST, ASXL1, SIRT4, ACTC1, TAF5, SLC7A6, CYP26B1, CCDC69, GADD45G, C1S, ATP1B4, MGP).

### 3.5 PPI network of hub genes

**FIGURE 6:**
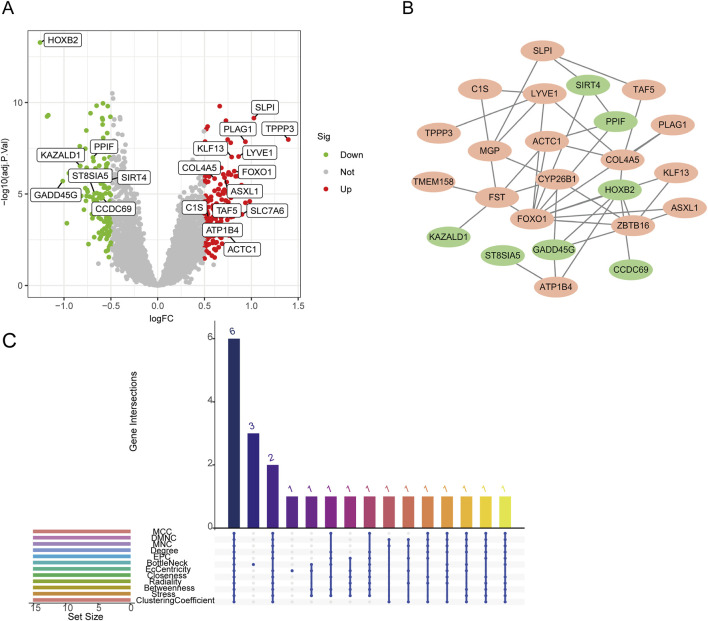
Identification of key genes. **(A)** The volcano plot depicts the expression levels of 25 key genes in sarcopenia patients in comparison to the control group. **(B)** The PPI network comprises these 25 central genes. **(C)** Upset plot of 12 topological algorithms determined by protein interaction network analysis.

Figure 6A illustrates the distinct expression profiles of 25 key genes in individuals with sarcopenia compared to control participants, as represented in a volcano plot.We used the Cytoscape plugin cytoHubba to perform centrality analysis on the PPI network composed of 25 hub genes (Figure 6B). The intersection of all twelve ranking algorithms integrated by Cytohubba revealed that FOXO1, ZBTB16, HOXB2, LYVE1, MGP, and CYP26B1 were identified as key genes for further analysis (Figure 6C).

### 3.6 Diagnostic performance and SHAP-based interpretability of core biomarkers

**FIGURE 7:**
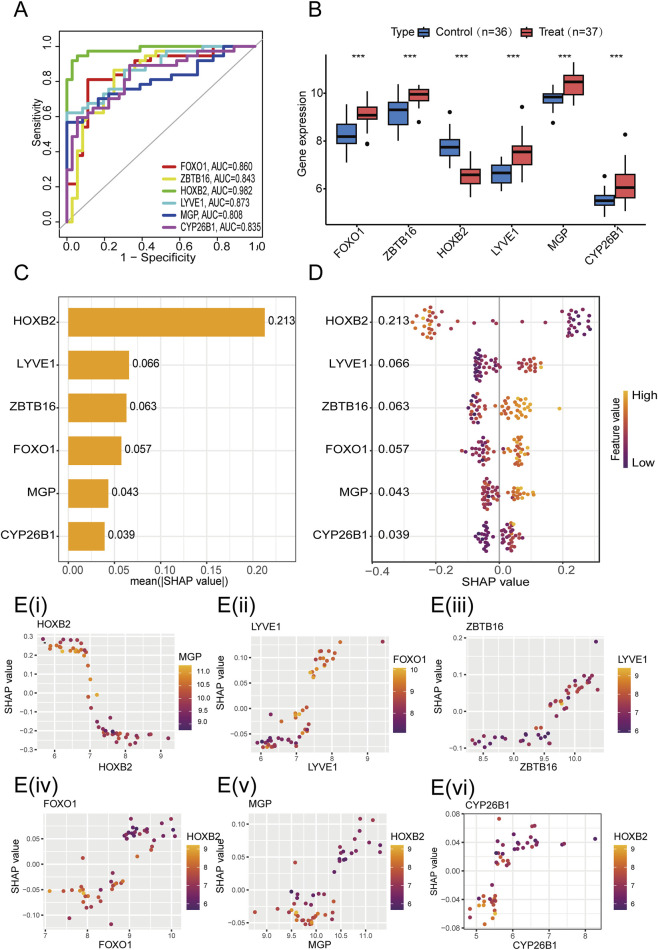
Gene expression characteristics and SHAP-based interaction analysis of the model. **(A)** ROC curves for individual model genes, demonstrating their discriminative performance in distinguishing sarcopenia from normal samples. **(B)** Box plots comparing gene expression levels between sarcopenia and control groups (blue: Control; red: Sarcopenia). Statistical significance is indicated by asterisks (***P < 0.001). **(C)** SHAP feature importance plot ranking genes according to their mean absolute SHAP values. **(D)** SHAP beeswarm plot illustrating the distribution of SHAP values for each gene across all samples; colors represent gene expression levels (orange: high, purple: low), indicating how expression magnitude influences model predictions. **(E)** SHAP dependence plots **(i–vi)** showing the non-linear relationships between individual gene expression levels and their SHAP values, with color gradients indicating potential interaction effects with secondary genes.

Single-gene ROC analysis indicated that the AUCs for FOXO1, HOXB2, ZBTB16, LYVE1, MGP, and CYP26B1 were 0.860, 0.982, 0.843, 0.873, 0.808, and 0.835, respectively, with HOXB2 showing an AUC >0.98, demonstrating excellent discriminative power (Figure 7A). The analysis of gene expression demonstrated a notable upregulation of the FOXO1, ZBTB16, LYVE1, MGP, and CYP26B1 genes within the sarcopenia cohort. Conversely, the HOXB2 gene exhibited a significant downregulation in this same group, thereby underscoring their possible involvement in the pathology of the disease (Figure 7B). The SHAP bee swarm plot and bar plot illustrated the impact of six key genes on model predictions. HOXB2 exhibited the highest average absolute SHAP value (0.213) and showed negative regulation, indicating its dominant role in discrimination, but increased expression was inversely related to predicted risk. LYVE1, ZBTB16, and FOXO1 showed positive SHAP values (0.057–0.066), indicating that higher expression levels of these genes were linked to an elevation in the predicted outcomes. MGP and CYP26B1 had weaker effects (SHAP <0.045) (Figures 7C,D). SHAP dependence plots revealed distinct non-linear and dose-dependent relationships between gene expression levels and their contributions to model output. Specifically, the negative contribution of HOXB2 plateaued at higher expression levels, suggesting a saturation effect (Figure 7Ei), while FOXO1 exhibited a transition from a negative to a positive impact as its expression increased, indicating a threshold-dependent shift in its influence on model prediction (Figure 7iv). Similar non-linear patterns were also observed for LYVE1, ZBTB16, MGP, and CYP26B1 (Figure 7Eii, iii, v, vi), further supporting the robustness of SHAP-based interpretability in revealing complex gene–model interactions.

### 3.7 Immune infiltration analysis and its association with diagnostic features

**FIGURE 8:**
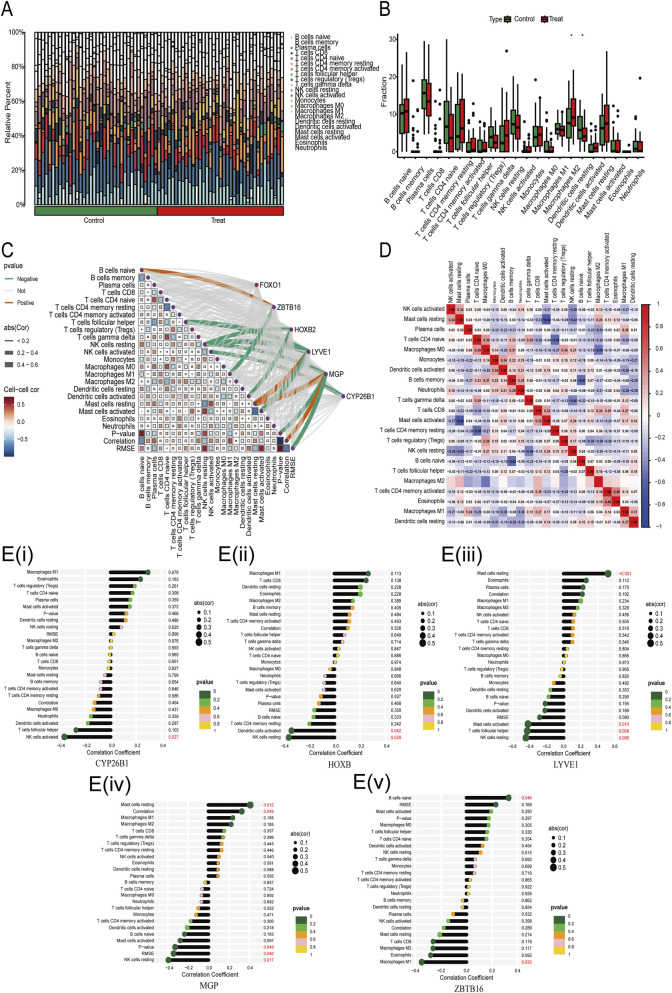
Research on immune characteristics related to sarcopenia and immune infiltrating cells associated with central genes. **(A)** Depicts a heatmap representing various immune cell types. **(B)** Presents a comparative analysis of the types of immune cell infiltration observed between the sarcopenia cohort and the control group. **(C)** Heatmap visualization illustrating the interaction landscape between the six model genes and 22 immune cell subsets. **(D)** Correlation matrix revealing the co-expression patterns among significant immune cells within the training cohort. **(E)** Lollipop plots illustrating the correlation between hub gene expression and immune cell infiltration levels in the training cohort. Each subpanel represents one core gene: **(E) (i)** CYP26B1, **(E) (ii)** HOXB2, **(E) (iii)** LYVE1, **(E) (iv)** MGP, **(E) (v)** ZBTB16.The horizontal axis indicates the correlation coefficient, and the dot size reflects the absolute value of the correlation. Dot colors represent the statistical significance (*P-value*), with warmer colors indicating stronger significance.

We employed the CIBERSORT algorithm to deconstruct the immunological landscape, quantifying the relative proportions of 22 infiltrating immune cell types across both sarcopenia and control samples (Figure 8A). Subsequent comparative analysis using the Wilcoxon test highlighted distinct immunological signatures, specifically revealing significant disparities in the abundance of M2 Macrophages and resting Dendritic cells between the two cohorts (Figure 8B). To further elucidate the interplay between molecular drivers and the immune microenvironment, we constructed correlation network heatmaps linking core genes to specific immune subsets (Figure 8C) and analyzed intra-immune interactions (Figure 8D). Notably, genes such as HOXB2, LYVE1, MGP, and CYP26B1 exhibited significant negative associations with resting NK cells, while ZBTB16 revealed a negative association with M1 macrophages (Figure 8E). These patterns strongly imply that macrophage and NK cell dynamics are central to the immune-regulatory pathways modulated by these hub genes.

### 3.8 MR validated the causal role of CYP26B1 in sarcopenia

**FIGURE 9:**
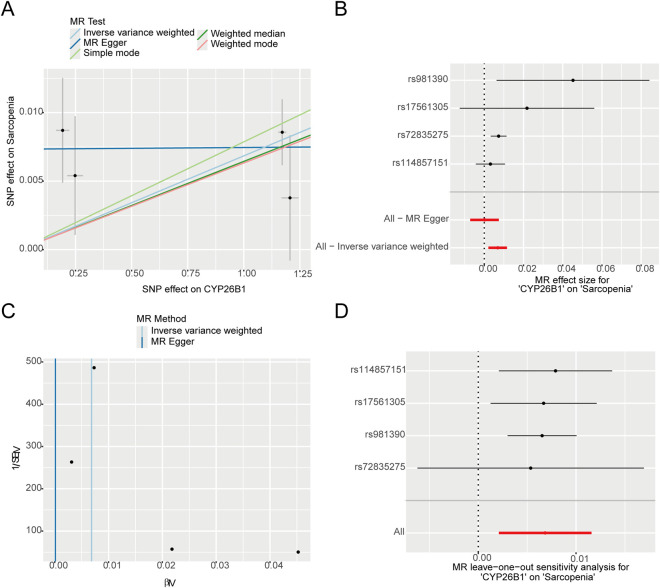
The causal relationship between CYP26B1 and sarcopenia risk. **(A)** Scatter plot illustrating the genetic associations of individual SNPs with CYP26B1 expression (exposure) versus sarcopenia risk (outcome). Regression slopes represent causal estimates from five distinct MR methods, including IVW and MR-Egger. **(B)** The forest plot showcases the levels of SNPs related to CYP26B1 and their impact on sarcopenia risk, along with the overall causal estimates (b, 95% confidence interval) obtained through MR-Egger and IVW techniques. **(C)** Funnel plot utilized to visualize potential heterogeneity and screen for directional pleiotropy among the genetic instruments. **(D)** The leave-one-out sensitivity analysis plot assesses the influence of each individual SNP on the causal estimates.

To probe the causal underpinnings of sarcopenia, we established a two-sample MR framework, mining the IEU Open GWAS database for valid SNPs associated with our six candidate genes (FOXO1, HOXB2, ZBTB16, LYVE1, MGP, and CYP26B1).By applying stringent instrumental variable criteria (*P* < 5e-8), we successfully isolated robust genetic instruments.The MR analysis demonstrated a noteworthy positive causal relationship between the expression levels of CYP26B1 (Odds Ratio = 1.006, 95% Confidence Interval: 1.002–1.011, *P* = 0.004) and the likelihood of developing sarcopenia (Figure 9A). While MR-Egger regression and pattern-based analyses yielded consistent directional outcomes, they did not achieve statistical significance. The heterogeneity assessments (Inverse Variance Weighted Q = 5.46, *P* = 0.14; MR-Egger Q = 1.18, *P* = 0.552) and pleiotropy evaluations (Egger intercept = 0.007, *P* = 0.174) revealed no significant heterogeneity or pleiotropic effects. Furthermore, the leave-one-out sensitivity analysis (Residual Sum of Squares observed = 7.01, *P* = 0.451) substantiated that no individual SNP exerted a considerable impact on the overall results, thereby reinforcing the reliability of these findings (Figures 9B–D).

### 3.9 *In vitro* model

**FIGURE 10:**
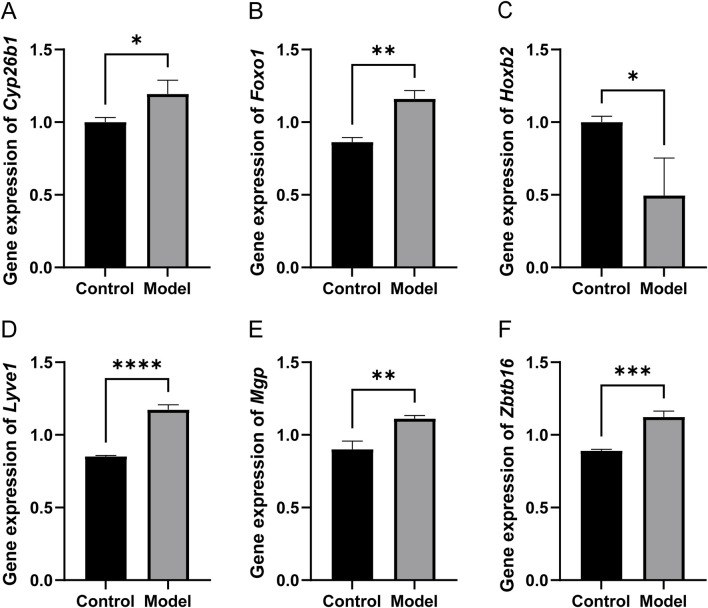
Altered expression of key genes in H_2_O_2_-induced C2C12 cell sarcopenia model. **(A)** Cyp26b1; **(B)** Foxo1; **(C)** Hoxb2; **(D)** Lyve1; **(E)** Mgp; **(F)** Zbtb16. *P < 0.05, **P < 0.01, ***P < 0.001, ****P < 0.0001.

To validate the reliability of the bioinformatic screening and the causal relationships identified through Mendelian randomization, we established an *in vitro* model of sarcopenia using C2C12 mouse myoblasts. As illustrated in Figures 10A–F, the mRNA expression levels of the six hub genes were quantified via qPCR. Compared with the Control group, the expression levels of Cyp26b1 (Figure 10A, *P* < 0.05), Foxo1 (Figure 10B, *P* < 0.01), Lyve1 (Figure 10D, *P* < 0.0001), Mgp (Figure 10E, *P* < 0.01), and Zbtb16 (Figure 10F, *P* < 0.001) were significantly elevated in the Model group. Conversely, the expression of Hoxb2 (Figure 10C, *P* < 0.05) exhibited a significant decrease following induction. These experimental results are highly consistent with the mRNA expression profiles identified in the GEO datasets (GSE1428 and GSE8479) and the machine learning predictions, confirming that these genes are key regulatory nodes in the progression of sarcopenia and providing a solid molecular basis for further mechanistic exploration.

## 4 Discussion

Sarcopenia is characterized by the gradual loss of skeletal muscle mass and strength, which poses significant challenges to public health, particularly among the elderly population (Chan and Yang, 2022; Wu et al., 2023; Yuan and Larsson, 2023). Epidemiological studies indicate that the prevalence of sarcopenia is 5%–13% in individuals aged 60–70 years, and it can be as high as 50% in those over 80 years, leading to increased morbidity, disability, and mortality (Ates Bulut et al., 2017; Kitamura et al., 2020; Milewska et al., 2022; Wu et al., 2023). However, the early symptoms of the disease are not obvious and can easily be overlooked. Furthermore, the clinical manifestations can vary among patients, complicating the effective study of sarcopenia progression (Xie et al., 2021). Although several biomarkers associated with sarcopenia have been identified, early diagnosis remains a significant challenge due to the complex underlying pathophysiological mechanisms and atypical early clinical presentations (Kalinkovich and Livshits, 2015; Xie et al., 2021; Veronesi et al., 2024). Therefore, developing accurate molecular biomarkers for identification and predictive diagnostic models is of utmost clinical significance for the early recognition and management of sarcopenia, as this may facilitate timely interventions and improve patient outcomes.

Our integrated analysis indicates that genes associated with sarcopenia mainly converge on extracellular matrix remodeling, muscle fiber organization, immune regulation, and retinoid metabolism. These biological processes are highly consistent with the core pathological features of sarcopenia, including impaired muscle structural integrity, dysregulated regeneration, chronic low-grade inflammation, and metabolic imbalance, suggesting that sarcopenia is a multifactorial disorder rather than the result of a single molecular abnormality (Wang et al., 2023; Cheng Y. et al., 2025; Damanti et al., 2025; Li N. et al., 2025).

Specifically, alterations in cell–matrix adhesion and contractile fiber assembly reflect disruption of cytoskeletal and extracellular matrix architecture that is essential for maintaining muscle strength and regeneration (Cruz-Jentoft and Sayer, 2019; Sun et al., 2025; Granic et al., 2026). In parallel, the enrichment of immune-related pathways highlights the contribution of immune dysregulation and persistent inflammatory signaling to muscle degeneration (Wilson et al., 2017; Xie et al., 2025). Moreover, the involvement of retinoid metabolism suggests that abnormalities in vitamin A and retinoic acid signaling may impair myogenic differentiation and tissue repair, providing an important metabolic link to sarcopenia pathogenesis (El Haddad et al., 2017; Kitakaze et al., 2023; Fang et al., 2024; Song et al., 2024; Cheung et al., 2025).

Among the six key genes confirmed in this study, genes such as Forkhead box O1(FOXO1) (Maimaiti et al., 2025), Homeobox protein Hox-B2 (HOXB2) (Chen Y. et al., 2024), Lymphatic vessel endothelial hyaluronan receptor 1 (LYVE-1) (Shen et al., 2024), and Matrix Gla protein (MGP) (Yang et al., 2025) have been supported by literature for their association with sarcopenia. Forkhead box O (FOXO) proteins are transcription factors that play a critical role in regulating skeletal muscle mass and control myogenic differentiation and fiber type specificity (Kamei et al., 2004; Huang et al., 2019). Studies have shown that in C2C12 cells and mouse models, β-sitosterol inhibits catabolic pathways by downregulating FoxO1, thereby preventing protein catabolism in skeletal muscle and reducing age-related muscle loss (Hah et al., 2022). Homeobox (Hox) genes are highly conserved transcription factors that play a crucial role in the proper development of muscles and the regulation of muscle-specific genes in mature muscle tissue (Houghton and Rosenthal, 1999; Poliacikova et al., 2021). As a member of this family, HOXB2 has been previously indicated to be downregulated in sarcopenia, consistent with the findings of this study (Chen Y. et al., 2024). Research has pointed out that after muscle injury, the expression of LYVE-1 is upregulated, leading to lymphangiogenesis, which promotes tissue repair and inflammation resolution (Taketa et al., 2024). A transcriptomic study on sarcopenia identified LYVE1 as a marker gene for tissue-resident macrophages in the skeletal muscle microenvironment, showing consistent expression across different age groups and pathological conditions (Shen et al., 2024). MGP regulates the calcification of bone and cartilage and is a substrate for γ-glutamyl carboxylase (Price et al., 1976). MGP is present in muscle, fat, and bone tissues, and all undergo vitamin K-dependent carboxylation modifications, coupling the degenerative processes of muscle, fat, and bone at the molecular level; its dysfunction may be a triggering and sustaining factor for multi-organ coordinated degeneration in sarcopenia (Schweighofer et al., 2022).

Additionally, this research identifies for the first time a correlation between Cytochrome P450 family 26 subfamily B member 1(CYP26B1) and Zinc finger and BTB domain containing 16 (ZBTB16) with this disease. ZBTB16 belongs to the evolutionarily conserved Zinc finger and BTB(ZBTB) transcription factor family (Cheng et al., 2021). Research has found that ZBTB16 expression in fibroblasts is critical for inflammation and fibrosis in the aging heart, a process modulated by macrophage mineralocorticoid receptors (Fraccarollo et al., 2024). Additionally, ZBTB16 has been reported to enhance the oligomerization of the inflammasome adaptor protein ASC by promoting its SUMOylation, thereby driving inflammasome assembly (Dong et al., 2023). All this evidence indicates that ZBTB16 may not only be a new candidate gene for sarcopenia, but also play a role in the development of the disease by regulating the chronic inflammatory process.

Compared with traditional sarcopenia biomarkers that mainly focus on single pathways such as inflammatory cytokines (e.g., tumor necrosis factor-α (TNF-α), interleukin-6 (IL-6), and interleukin-8 (IL-8)) (Picca et al., 2022; Calluy et al., 2026; Sokolova et al., 2026) or muscle-specific proteins (e.g., muscle RING-finger protein-1 (MuRF1), FOXO1, eukaryotic translation elongation factor 1 epsilon 1 (EEF1E1), and perilipin-2 (PLIN2)) (Van Long et al., 2023; Dun et al., 2024), our six-gene signature (FOXO1, HOXB2, LYVE1, ZBTB16, MGP, and CYP26B1) provides a more comprehensive and multi-dimensional representation of the disease by integrating immune regulation, structural integrity, and metabolic homeostasis, which better reflects the multifactorial nature of sarcopenia (Grima-Terrén et al., 2024; Sato et al., 2024). Methodologically, this study goes beyond conventional differential expression analyses by integrating WGCNA, machine learning algorithms, PPI network construction, and SHAP interpretation, an analytical strategy that is increasingly adopted in recent high-impact bioinformatics-driven biomedical studies (Sackett et al., 2022; Chen B. et al., 2024; Niu et al., 2024; Wang et al., 2024; Liu et al., 2025). More importantly, we introduced Mendelian randomization to move from correlation to causality (Davey Smith and Hemani, 2014; Wu Q. et al., 2025), identifying CYP26B1 as a potential causal risk factor for sarcopenia. Together, these advances establish a causally informed, multi-pathway biomarker framework that offers clear advantages over existing single-dimension biomarkers.

The significantly elevated expression level of the CYP26B1 gene in sarcopenia patients warrants further exploration of its molecular mechanisms in muscle metabolism regulation. CYP26B1 is a member of the cytochrome P450 enzyme family that metabolizes retinoic acid (RA) into easily degradable derivatives (Zhang Z. et al., 2025). RA is a bioactive derivative of vitamin A, which is particularly important in regulating many biological processes, such as cell differentiation, growth and apoptosis (Noy, 2010; Guo et al., 2022). For example, it is found that RA can promote myoblasts to differentiate into mature muscle fibers by antagonizing TGF-β signal mediated by C/EBPβ, thus enhancing muscle production ability (Lamarche et al., 2015; Takaya et al., 2022). CYP26B1 is crucial in the metabolic pathway of vitamin A, as it facilitates the conversion of surplus retinoic acid into its inactive polar derivatives (Khosasih et al., 2023). Vitamin A is essential for cell development and tissue homeostasis, and studies have shown that it can stimulate muscle cell differentiation and promote skeletal muscle repair, playing an important role in maintaining muscle function (Zhang et al., 2023; Fang et al., 2024). However, elevated CYP26B1 expression may lead to excessive metabolism of RA, resulting in insufficient local active signaling, thereby weakening the beneficial effects of vitamin A on muscle maintenance and repair. Although previous studies have suggested that abnormal vitamin A metabolism may be associated with an elevated risk of sarcopenia, the precise molecular mechanisms of CYP26B1 in this process remain unclear. Additionally, its potential clinical value as a biomarker or therapeutic target necessitates further investigation and validation (Fang et al., 2024).

The MR analysis of our study found that there was an obvious positive causal relationship between the expression level of CYP26B1 and the risk of sarcopenia. This finding is very similar to other reports. For example, in tumors and Cardiac Developmental Abnormalities, the expression of CYP26B1 also increased, and it will affect cell proliferation and differentiation by degrading retinoic acid, which has been confirmed in (Ahuja et al., 2022; Gu et al., 2025). It has been reported that activating Wnt/β-catenin pathway can decrease the expression of CYP26B1, so that the signal of retinoic acid will be enhanced, thus promoting osteogenesis and tissue regeneration (Yao et al., 2023). In sarcopenia patients and elderly mouse models, knocking down STAT3 with siRNA can activate the Wnt/β-catenin pathway, enhancing the expression of myogenic markers and restoring muscle mass and function (Deng et al., 2024; Zhang S. et al., 2025). However, the abnormal upregulation of CYP26B1 may interfere with this pathway, leading to weak differentiation signal and muscle production problems.

In order to study the relationship between key genes and the immune microenvironment of sarcopenia more carefully, we used the method of CIBERSORT to analyze how immune cells entered the muscle. Many studies have told us that inflammation and abnormal immune system have a great influence on the occurrence of sarcopenia (Saini et al., 2016; Zhang et al., 2022; Zhou et al., 2025). The aging of our immune system will make our muscles age in several ways, and finally lead to sarcopenia (Wang et al., 2019). Additionally, scientists conducted genetic analysis and tissue examination on patients with sarcopenia, and found that in the microenvironment of human skeletal muscle, the types of immune cells, how they stay together and how they interact with each other have changed with people getting older. These changes make the elderly or people with sarcopenia prone to a long-term inflammation in their muscles (Shen et al., 2024). Our study indicates that the genes HOXB2, LYVE1, MGP, and CYP26B1 show significant negative correlations with NK cells resting. NK cells, as an important component of the innate immune system, play a key role in maintaining immune homeostasis, clearing abnormal cells, and regulating the tissue microenvironment (Lutz and Quinn, 2012; Crinier et al., 2020; Vivier et al., 2024). ZBTB16 demonstrates an inverse correlation with M1 macrophages. Macrophages are essential in tissue regeneration and the maintenance of muscle homeostasis, as they release growth factors and cytokines that regulate the behavior of muscle stem cells and the activation of myofibroblasts (Chen et al., 2022). However, at present, there is still a lack of systematic research on the molecular mechanisms by which these key genes (HOXB2, LYVE1, MGP, CYP26B1, and ZBTB16) regulate the functional states of NK cells and macrophages in the context of sarcopenia. The results of this study mainly reflect the statistical correlations between gene expression levels and immune cell infiltration, and thus cannot directly infer causal regulatory relationships. Further functional experiments and mechanistic studies are required to elucidate the related molecular pathways and regulatory networks, which will contribute to a more comprehensive understanding of the role of immune regulation in the development and progression of sarcopenia.Moreover, whether these genes interact with each other in a coordinated or synergistic manner to modulate immune cell behavior and muscle microenvironment remodeling remains unknown and warrants further investigation.

This study has made progress in identifying and functionally predicting key genes related to sarcopenia; however, several limitations should be acknowledged. First, the diagnostic models were mainly based on transcriptomic data from public GEO datasets and lacked validation in independent clinical cohorts, which may limit their generalizability. Second, although MR analysis suggested a causal role of CYP26B1 in sarcopenia, this conclusion still requires validation through functional experiments. In addition, while the six-gene panel showed strong combined diagnostic performance, their potential biological synergistic interactions were not directly explored. Moreover, due to the absence of detailed clinical information in the GEO datasets, potential confounding factors such as Body mass index, lifestyle, comorbidities, and medication use could not be fully adjusted for, which may have influenced the observed associations.

Future studies should integrate spatial transcriptomics and single-cell sequencing, together with functional experiments such as gene knockdown or overexpression, to clarify causal mechanisms and gene–immune cell interactions. In addition, multi-center clinical cohorts with comprehensive clinical and lifestyle covariates are needed to validate the multi-gene diagnostic model and to explore possible synergistic regulatory effects among these genes.

## 5 Conclusion

This study successfully combined WGCNA and multiple machine learning techniques to identify six key sarcopenia biomarkers: HOXB2, FOXO1, LYVE1, ZBTB16, MGP, and CYP26B1, with PCR confirmation in cell models. Most significantly, our MR analysis identified CYP26B1 as a causal factor, linking retinoic acid metabolism directly to sarcopenia pathogenesis. Theoretically, this completes the chain from genetic association to immune-metabolic causation. Clinically, while the six-gene panel aids diagnosis, CYP26B1 represents a novel therapeutic target for modulating retinoic acid pathways and the immune microenvironment.

## Data Availability

The datasets presented in this study can be found in online repositories. The names of the repository/repositories and accession number(s) can be found in the article/Supplementary Material.
